# Small dose rate changes significantly affect the magnitude of cellular signaling in response to high LET exposure

**DOI:** 10.1093/jrr/rrt203

**Published:** 2014-03

**Authors:** D.M. Sridharan, R.D. Roppel, R. Chan, W.C. Wilson, M.K. Whalen, L.J. Chappell, J.M. Pluth

**Affiliations:** 1 Lawrence Berkeley National Laboratory, Life Sciences Division, 717 Potter Street, Berkeley, CA 94710, USA; 2 NASA, Lyndon B. Johnson Space Center, 2101 NASA Parkway, Houston, TX, USA

**Keywords:** mammary epithelial, phospho-protein signaling, ATF2, genomic instability, cancer risk

## Abstract

Purpose: The purpose of this study was to determine whether small differences in dose rate influence cellular response following radiation exposure.

Introduction: Astronauts will receive chronic low-dose exposure to charged particles during space travel. The heterogeneous mix of energetic heavy ions in the space radiation environment poses a major challenge to understanding the biological significance of these exposures. Estimating the relative risk for carcinogenesis from high LET (linear energy transfer) exposures and identifying ways to mitigate these risks are key to ensuring the future safety of astronauts. Several factors complicate the accurate modeling of cancer risk from these exposures. In addition to dose effects, the rate at which dose is delivered plays a key role in the biological consequences of high LET exposure. Low-dose rate studies thus far have assessed differences between acute doses of a couple of minutes in comparison with prolonged exposures that are delivered over the course of a day or longer. Our studies aim to understand if small differences in dose rate delivered between 0.5 and 2 min show any biological effect. In these studies, we have utilized ATM-mediated phosphorylation of ATF2 in response to radiation exposure as a tool to evaluate dose rate effects. As mammary tissues are radiation-sensitive and are of particular interest to NASA, our studies were carried with isogenic mammary epithelial and fibroblasts cells.

Materials and methods: Normal human primary mammary epithelial and fibroblast cells were initiated 1 week prior to X-ray or Fe 1000 MeV/u exposure. Cells at ∼80% confluency on the day of irradiation were irradiated at room temperature. Cells were fixed and stained at early time points (0.5–24 h) following different dose exposures for flow cytometry analysis of phospho-ATF2 as described previously [ 1]. The mean fold induction of pATF2 fluorescence in cells in the G1 phase of cell cycle was determined relative to control samples.

Results: Small dose rate changes, ∼2–2.5 fold change, resulted in obvious changes in the levels of phospho-signaling observed. The earliest time point (0.5 h) revealed the greatest effect from small dose rate changes, with most differences eliminated by 24 h post-exposure. Different cell types revealed differences in the effects observed with fibroblasts showing a greater induction with higher dose rate and epithelial cells showing a greater induction post-low-dose rate exposures. An example of the notable difference in phospho-protein induction observed in the fibroblast strain when even a minor difference in dose rate is used (2-2.5 fold) is shown in Fig. 1.

Conclusion: The phospho-signaling profile for a key DNA damage-response protein, pATF2, was measured at both early and late (0.5–24 h) time points. Our studies reveal that even small dose rate changes show significant differences in terms of the levels of phospho-protein induction. Cell type is a major determinate in this effect, and could predict the late consequences of exposure.

**Fig. 1.**
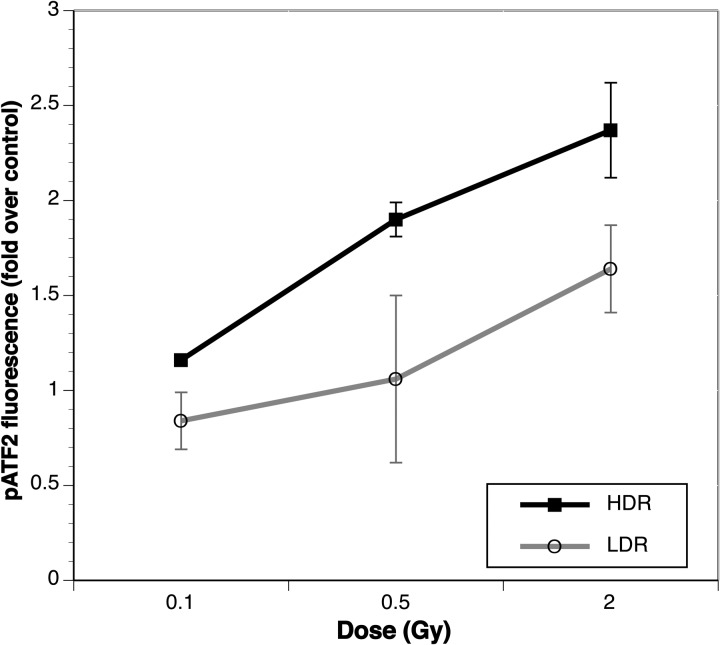
Comparison of phospho ATF2 induction in a primary fibroblast line (184 Fb) upon exposure to a high dose rate (HDR) vs a low dose rate (LDR) of Fe 1000 MeV/u. A notable difference is observed at all doses even though the difference between dose rates is very minor (2-2.5 fold).

Comparison of phospho ATF2 induction in a primary fibroblast line (184 Fb) upon exposure to a high dose rate (HDR) vs a low dose rate (LDR) of Fe 1000 MeV/u. A notable difference is observed at all doses even though the difference between dose rates is very minor (2-2.5 fold).
